# Colin Blakemore (1944–2022)

**DOI:** 10.1017/S0952523822000074

**Published:** 2023-02-09

**Authors:** Lothar Spillmann

**Affiliations:** Department of Neurology, University of Freiburg, Freiburg, Germany

Colin Blakemore, who died in Oxford on June 27 last year at the age of 78, was a world-renowned British neuroscientist and a highly influential and much-admired member of the vision community.

As a medical student at Cambridge, Blakemore was influenced by Richard Gregory, and he subsequently maintained a keen interest in all aspects of visual science. He is best remembered for his studies on the development of the visual brain in kittens and the demonstration of neural plasticity. His findings were crucial for a better understanding of how brain cells organize themselves in response to the visual environment after birth.

After graduating with a First at Cambridge, Blakemore went to the University of California at Berkeley in 1965 for his Ph.D. There he worked with Horace Barlow and Jack Pettigrew on binocular depth discrimination in the cat. He found that the response of binocular units in area V1 depended crucially on the alignment of the binocular stimulus in the two eyes. When the stimulus in one eye was off target, the response was vetoed. Blakemore returned to Cambridge in 1968 to take up a lectureship in physiology and, 3 years later, to become a Fellow at Downing College. It was during that time that he left the study of perception behind in favor of combining behavioral methods and neurophysiological techniques for the study of the visual system.

In a ground-breaking experiment with Grahame Cooper, in 1970, he demonstrated that a kitten, which was reared in complete darkness since birth and then exposed to a vertically striped cylinder for 5 hours every day, was severely visually impaired when tested half a year later. In addition to showing no placement response and being seemingly oblivious toward an approaching object, the kitten behaved as if it was blind to a moving horizontal line. Conversely, a kitten that had been exposed to a horizontally striped cylinder, was blind to a moving vertical line. These results showed that the striate cortex could be modified by selective experience early in life and that normal visual experience is crucial for normal maturation. When the authors recorded from cortical cells, the typical orientation tuning was gravely disturbed and only those cells tuned to near-vertical (or horizontal) responded, consistent with the behavioral deficit.

This experiment triggered the great Nature–Nurture debate in the seventies and eighties. Numerous studies were performed in Cambridge and by other vision scientists, to further elucidate the early development of vision and visual perception. In the early 1970s, for example, Blakemore and Richard Van Sluyters embarked on a series of deprivation studies in kittens, in which they surgically closed the lids of one eye and showed that the normal binocular dominance of cortical cells shifted entirely to the other eye. Conversely, when the previously open eye was closed and the initially closed eye reopened, the ocular dominance was reversed, so that now every cell was dominated by inputs from the initially deprived eye. Importantly, this only worked within a critical period of up to 3 months, with a peak at about 30 days.

Blakemore together with Anthony Movshon and Van Sluyters went one step further by exposing kittens to a grating of a certain spatial frequency and found that they could bias cortical cells to that frequency. Thus, the response of neurons could be modified by selective exposure to the spacing of the grating stripes. The importance of these results and those of Torsten Wiesel at Harvard Medical School, who had studied kittens and monkeys with surgically induced squint, was immediately recognized by clinical ophthalmologists such as Gunter von Noorden. They had long tried to understand the development of amblyopia in strabismic children, a condition in which the visual resolution and contrast sensitivity in one eye is irreversibly impaired due to the misalignment of the two eyes. Based on these results, eye surgeons from around the world now perform corrective surgery in squinting children before the age of 4, that is, within the critical period for human vision.

Blakemore also showed that when kittens were reared with a diffuser in front of their eyes, thereby blurring the retinal image, cortical cells became unresponsive. This explains why children born with congenital cataract (i.e., a cloudy lens) become amblyopic or blind due to a lack of pattern vision early in life.

Blakemore was a singularly gifted speaker, who communicated his results and observations with elegance, flair, and charisma. It was therefore no surprise that at the age of 32, he became the youngest person to deliver the prestigious Reith lectures on BBC radio. The topic chosen was “Mechanics of the Mind.” Twelve years later, he also presented a 13-part BBC TV series on the *The Mind Machine.* This was the time when vision research was at apogee, when there was something new and exciting every other month, and when there were heroes such as Blakemore to look up to. Several books testify to his unique style also in print.

In 1979, at the age of 35, Blakemore was appointed as the Waynflete Professor of Physiology at the University of Oxford, and 13 years later, he was elected as a Fellow of the Royal Society. Researchers in his group, notably Frank Sengpiel, continued to pursue the topic of neural plasticity in his laboratory. In 2003, Blakemore became head of the Medical Research Council, which he reformed within 4 years, substantially increasing the amount of research funding. Because of his defense of animal experiments as well as his own experimental program, from the 1980s, he had been savagely attacked by animal rights activists. These attacks included a parcel bomb addressed to his children and delivered to his home but luckily spotted before detonation. But he stood his ground and, thanks largely to his tireless communication and public engagement, the tide of public opinion turned. In 2007, he returned to Oxford and subsequently to the University of London and then the City University of Hong Kong. He was knighted by the Queen in 2014. Blakemore was honored for his scientific achievements in many academies and societies.

Blakemore was a long-distance runner and in superb physical shape well into his seventies. He completed 18 marathons. Thus, we were shocked to learn in 2021 that he had been diagnosed with Amyotrophic Lateral Sclerosis, a crippling and fatal neuromuscular disease. In a fitting tribute, on August 7th of last year, Oxford University staged a one-day symposium in his honor and renamed the Large Lecture Hall after him. The dedication reads:

The Large Lecture Theatre in the Sherrington building is being renamed the Blakemore Lecture Theatre in recognition of the sustained and long-standing contribution of Professor Sir Colin Blakemore FRS as Waynflete Professor of Physiology and his time as an outstanding lecturer for generations of medical students and physiology students on the wonders of the brain.

Tragically, Colin’s wife Andrée, whom he had met when they were 15, died 5 months before him. They are survived by their daughters, Sarah-Jayne, Professor of Psychology at Cambridge, Sophie, and Jessica. Colin Blakemore was a visionary, a brilliant colleague, and a good friend. He will be missed.

